# Analysis of avian influenza A (H3N8) viruses in poultry and their zoonotic potential, China, September 2021 to May 2022

**DOI:** 10.2807/1560-7917.ES.2023.28.41.2200871

**Published:** 2023-10-12

**Authors:** Pengfei Cui, Jianzhong Shi, Cheng Yan, Congcong Wang, Yuancheng Zhang, Yaping Zhang, Xin Xing, Yuan Chen, Jie Zhang, Liling Liu, Xianying Zeng, Guobin Tian, Chengjun Li, Yasuo Suzuki, Guohua Deng, Hualan Chen

**Affiliations:** 1 State Key Laboratory for Animal Disease Control and Prevention, Harbin Veterinary Research Institute, CAAS, Harbin, China; 2 National Poultry Laboratory Animal Resource Center, Harbin Veterinary Research Institute, CAAS, Harbin, China; 3 These authors contributed equally to this manuscript; 4 Western Research Institute, CAAS, Changji, China; 5 Department of Medical Biochemistry, University of Shizuoka School of Pharmaceutical Sciences, Shizuoka, Japan

**Keywords:** Avian influenza virus, H3N8, evolution, Guinea pig, transmission

## Abstract

**Background:**

Two human cases of avian influenza A (H3N8) virus infection were reported in China in 2022.

**Aim:**

To characterise H3N8 viruses circulating in China in September 2021−May 2022.

**Methods:**

We sampled poultry and poultry-related environments in 25 Chinese provinces. After isolating H3N8 viruses, whole genome sequences were obtained for molecular and phylogenetic analyses. The specificity of H3N8 viruses towards human or avian receptors was assessed in vitro. Their ability to replicate in chicken and mice, and to transmit between guinea pigs was also investigated.

**Results:**

In total, 98 H3N8 avian influenza virus isolates were retrieved from 38,639 samples; genetic analysis of 31 representative isolates revealed 17 genotypes. Viruses belonging to 10 of these genotypes had six internal genes originating from influenza A (H9N2) viruses. These reassorted viruses could be found in live poultry markets and comprised the strains responsible for the two human infections. A subset of nine H3N8 viruses (including six reassorted) that replicated efficiently in mice bound to both avian-type and human-type receptors in vitro. Three reassorted viruses were shed by chickens for up to 9 days, replicating efficiently in their upper respiratory tract. Five reassorted viruses tested on guinea pigs were transmissible among these by respiratory droplets.

**Conclusion:**

Avian H3N8 viruses with H9N2 virus internal genes, causing two human infections, occurred in live poultry markets in China. The low pathogenicity of H3N8 viruses in poultry allows their continuous circulation with potential for reassortment. Careful monitoring of spill-over infections in humans is important to strengthen early-warning systems and maintain influenza pandemic preparedness.

**Key public health message**

**What did you want to address in this study?**

Avian H3N8 viruses constitute an influenza virus subtype mainly detected in birds. In birds, H3N8 viruses usually cause little or no sign of disease. Spill-over infections to people nonetheless sporadically happen and two human H3N8 viral infections were reported in China in 2022. We wanted to investigate H3N8 viruses circulating in poultry and poultry-related environments in China in September 2021−May 2022.

**What have we learnt from this study?**

We isolated H3N8 viruses from samples collected in various Chinese provinces. In laboratory assays, some of the viruses could bind to both human- and avian-type receptors. Further analyses revealed that a number of viruses, including those having caused human infections, had acquired genes from other influenza virus subtypes called H9N2. These viruses transmitted between guinea pigs via respiratory droplets.

**What are the implications of your findings for public health?**

Our findings shed more light on how avian H3N8 influenza viruses can evolve in poultry by adopting genes from H9N2 influenza viruses, which are also found in poultry. The results increase our understanding of how H3N8 viruses can infect mammals and potentially pose a human health threat. Careful monitoring of human H3N8 viral infections is important for influenza pandemic preparedness.

## Introduction

Influenza A viruses are important zoonotic pathogens that continue to evolve and challenge animal and human health. The Influenza A(H1N1), (H2N2), and (H3N2) viruses have caused four influenza pandemics in humans since 1918 [1]. In recent decades, viruses of the H5 and H7 subtypes have caused avian influenza outbreaks in poultry and wild birds and severe human infections and deaths in multiple countries [2-6]. Active control strategies implemented in poultry, including culling and vaccination, have largely prevented or eliminated human infections with the H5 and H7 avian influenza viruses [7]. Nevertheless, several subtypes of low pathogenic avian influenza viruses, including H6N1, H9N2, H10N3, and H10N8, have crossed the species barrier and caused human infections in China [8-10]. Because low pathogenic avian viruses usually do not cause disease or death in animals, however, they constitute a low priority for animal disease control, allowing their unmonitored evolution in nature.

On 25 April 2022, a 4-year-old boy infected with H3N8 avian influenza virus was reported in the Henan province of China, constituting the first case of human infection with the avian influenza A (H3N8) virus [11]. The following month, a 5-year-old child infected with H3N8 virus was reported in Changsha, the capital of Hunan province [12]. The objective of this study was to investigate the origin of H3N8 viruses that had infected people. Another aim was to understand H3N8 virus evolution and diversification in nature and whether this may pose any potential threat to public health.

## Methods

We performed a detailed study of H3N8 viruses isolated in China between September 2021 and May 2022. The genome sequences of such viruses were subjected to molecular characterisation and phylogenetic analyses and their receptor-binding properties assessed in vitro. We also investigated their replication, virulence, and transmissibility in different animals.

### Virus isolation

Samples collected during active surveillance were processed in the enhanced biosafety level 2 (BSL2 +) facility at the Harbin Veterinary Research Institute of the Chinese Academy of Agricultural Sciences (HVRI, CAAS). Swab samples from animals were individually inoculated into 10-day-old embryonated chicken eggs and incubated for 48 hours at 37 °C. The haemagglutinin (HA) subtype of the isolates was identified by using the HA inhibition (HI) test and the neuraminidase (NA) subtype of the isolates was confirmed by direct sequence analysis. Thirty-one of the H3N8 viruses sequenced in this study were biologically cloned three times by limiting dilution in embryonated specific-pathogen-free (SPF) chicken eggs, and the virus stocks were grown in SPF chicken eggs and maintained at − 70 °C.

### Genetic and phylogenetic analysis

The genome of H3N8 viruses from 31 isolates was sequenced on an Applied Biosystems DNA Analyzer (3500xL Genetic Analyzer, United States (US)). The obtained nucleotide (nt) sequences were deposited in GISAID databases (https://www.gisaid.org) under accession numbers EPI2454581–EPI2454828. The nt sequences were edited with the SeqMan module of the DNAStar package. The time-scaled phylogenetic tree of the HA gene was inferred by using an asymmetric continuous-time Markov chain with Bayesian stochastic search variable selection implemented in Bayesian Evolutionary Analysis Sampling Trees (BEAST; v1.10.4). The phylogenetic analysis of NA and the six internal genes was performed by using the Molecular Evolutionary Genetics Analysis (MEGA) 7.0.14 software package with the neighbour-joining method, and the tree topology was evaluated by 1,000 bootstrap analyses. We used 95% sequence identity cutoffs to categorise the gene groups in the phylogenetic trees.

### Receptor-binding assays

The receptor-binding specificity of the H3N8 viruses was analysed by using a solid-phase binding assay as described previously [13]. Two different glycopolymers, α-2,3-sialylglycopolymer (avian-type receptor) and α-2,6-sialylglycopolymer (human-type receptor), were used in this study. Chicken antiserum against A/chicken/Guangdong/S1286/2009(H3N8), A/black swan/Beijing/1/2021(H5N8), and A/Sichuan/1/2009(H1N1) virus were generated and used as the primary antibodies, and horseradish peroxidase (HRP)-conjugated goat-anti-chicken antibody (Sigma-Aldrich, St. Louis, MO, US) served as the secondary antibody.

### Mouse study

Twenty-four groups of eight 6-week-old female BALB/c mice (Beijing Experimental Animal Center, Beijing, China) were lightly anesthetised with CO_2_ and inoculated intranasally (i.n.) with 10^6^ 50% egg infectious dose (EID_50_) of H3N8 viruses in a volume of 50 μL. The nasal turbinate, lungs, spleen, kidneys, and brain of three infected mice in each group were collected on day 3 post-inoculation (p.i.) for virus titration in chicken eggs. The remaining five mice in each group were monitored daily for body weight loss and survival for 14 days.

### Replication and transmission of H3N8 viruses in guinea pigs

Hartley strain female guinea pigs weighing 300–350 g (Beijing Experimental Animal Center, Beijing, China) that were serologically negative for influenza viruses were used in this study. To investigate the replication of the virus, six groups of two guinea pigs were anesthetised with ketamine (20 mg/kg) and xylazine (1 mg/kg) and inoculated i.n. with 10^6^ EID_50_ of test virus in a 300 μL volume (150 μL per nostril). The guinea pigs were euthanised on day 3 p.i. and their nasal washes and lung tissues were collected for virus titration in eggs. 

The respiratory droplet transmission study for each test virus was performed with three ‘1:1’ pairs of guinea pigs as shown in Supplementary Figure S1. Briefly, six groups of three guinea pigs were inoculated i.n. with 10^6^ EID_50_ of test virus in a 300 μL volume (150 μL per nostril) and housed in three separate cages within a special isolator; 24 hours later, three naive guinea pigs in each group were placed in three adjacent cages (4 cm away). Nasal washes were collected every 2 days p.i. to detect virus shedding, and sera were collected from all animals on day 21 p.i. to assess seroconversion.

### Chicken studies

Four groups of 13 4-week-old SPF White leghorn chickens (National Poultry Laboratory Animal Resource Center, Harbin, China) housed in isolators were inoculated i.n. with 10^6^ EID_50_ of H3N8 virus in a volume of 0.1 mL. On day 3 p.i., three chickens in each group were euthanised and their organs, including larynxes, tracheas, lungs, hearts, livers, spleen, kidneys, pancreas, caecum, and brains were collected for virus titration in eggs. The oropharyngeal and cloacal swabs of the remaining 10 chickens were also collected on days 3, 5, 7, 9, and 11 p.i. to assess virus shedding. All chickens were observed for signs of disease or death for 14 days.

## Results

### Isolation of H3N8 avian influenza viruses

In total, 38,216 swab samples and 423 environment samples were collected at live poultry markets, poultry farms, and poultry slaughterhouses from 25 provinces in China between September 2021 and May 2022. From these samples, 1,044 avian influenza virus isolates of different subtypes were obtained, of which 98 were H3N8 viruses (Table 1). As shown in Table 1, 15 H3N8 virus isolates were derived from some of the 18,952 samples collected between September 2021 and December 2021, whereas 83 H3N8 virus isolates originated from some of the 19,687 samples collected between January 2022 and May 2022. Of note, 90 viruses, seven viruses, and one virus, respectively, were isolated from poultry markets, duck farms, and duck slaughterhouses (Table 1), indicating that the H3N8 viruses may have been circulating in duck farms and live poultry markets.

**Table 1:** Description of sampling of poultry or poultry-related environments for detection of avian influenza viruses and information on number and provenance of H3N8 viral isolates, China, September 2021−May 2022 (n = 38,639 samples collected)

Period	Location	Number of samples collected	Number of provinces investigated^a^	Number of H3N8 virus isolates		Provinces found with H3N8 virus	
				Total	Number per origin	Number	Names
Sep−Dec 2021	Market	8,223	19	9	6 from chicken	3	Fujian, Guangdong, Zhejiang
					3 from ducks		
	Farm	9,107	23	5	5 from ducks	2	Hubei, Jiangxi
	Slaughterhouse	1,622	9	1	1 from ducks	1	Zhejiang
Jan−May 2022	Market	9,422	16	81	61 from chicken	10	Anhui, Fujian, Guangdong, Guangxi, Guizhou, Henan, Hunan, Jiangsu, Ningxia, and Zhejiang
					14 from ducks		
					3 from geese		
					3 from the environment		
	Farm	9,584	23	2	2 from ducks	1	Anhui
	Slaughterhouse	681	5	0	Not applicable	0	Not applicable

^a^ A total of 25 provinces were investigated over the study period.

### Genetic analysis of the H3N8 viruses

**Figure 1:**
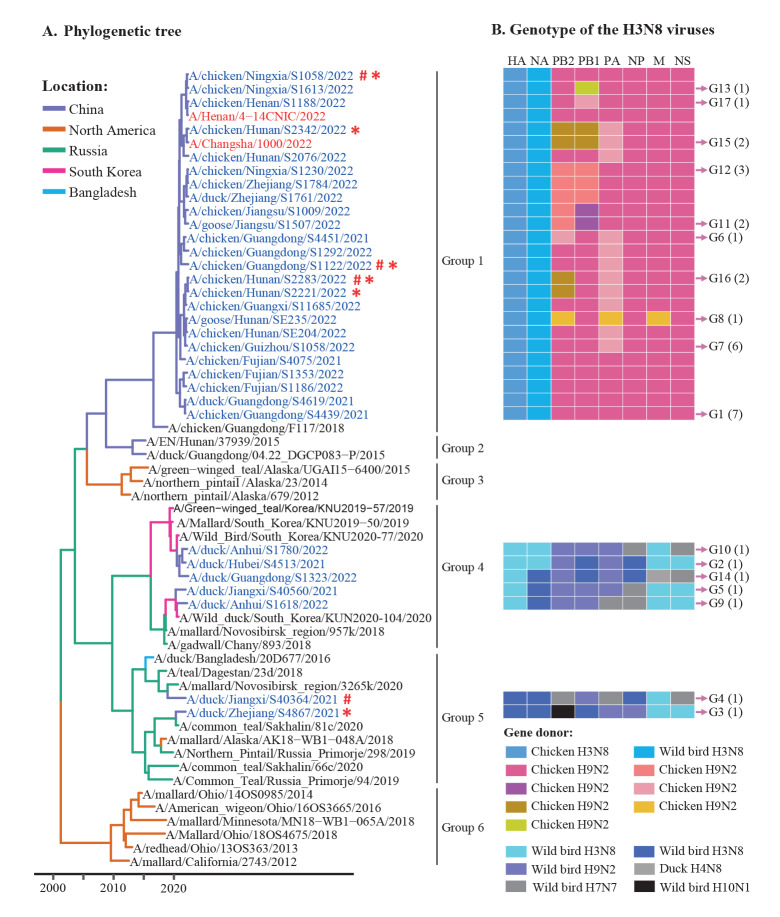
Phylogenetic analysis of H3N8 viral haemagglutinin sequences from different parts of the world, including two sequences from viruses that infected humans and 31 sequences retrieved in this study in September 2021−May 2022 in China (n = 59 sequences in total)

BEAST: Bayesian Evolutionary Analysis Sampling Trees; HA: haemagglutinin; NA: neuraminidase; PA: polymerase A; PB: polymerase B; M: matrix; NP: nucleoprotein; NS: non-structural protein.

To understand the genetic relationships of these H3N8 viruses, the genomes of 31 representative viruses from different sampling times, locations, and avian species (Table 2) were fully sequenced and compared with those reported by others. The time-scaled phylogenetic tree of the HA gene was inferred with the HA genes of 59 H3N8 viruses, including the 31 H3N8 viruses sequenced in this study, two H3N8 viruses that had infected humans in China, and 26 representative viruses whose genetic sequences were downloaded from the GISAID EpiFlu Database (information on the originating and submitting laboratories of the sequences, as well as on the sequences is shown in Supplementary Table S1). The HA gene of the 59 H3N8 viruses shared 80–99.9% identity at the nt level, forming six phylogenetic groups, and the HA genes of the 31 viruses sequenced in this study were assigned to groups 1, 4, and 5, respectively (Figure 1, panel A). These viruses had the same amino acid motif (-PEKQTR/GLF-) at their HA cleavage site, indicating that they are low pathogenic avian influenza viruses. The NA polymerase basic (PB)2, PB1, polymerase acidic (PA), nucleoprotein (NP), matrix (M), and non-structural (NS) genes of the 31 H3N8 viruses in this study shared 92.9–100%, 85.3–99.9%, 87–99.9%, 87.1%–100%, 88.9–100%, 87.9–100%, and 69.2–100% identity, respectively, and formed 3–8 groups in their phylogenetic trees, which are presented in Supplementary Figure S2.

**Table 2:** Sample and genotype information of the H3N8 viruses sequenced in this study as well as the two viruses that infected humans, China, September 2021−May 2022 (n = 33 viruses)

Sample information		Virus		
Date	Location	Full name	Abbreviation	Genotype^b^
14 Apr 2022	Hospital	A/Henan/4–14CNIC/2022^a^	A/HeN/4–14CNIC/22	***G1***
11 May 2022	Hospital	A/Changsha/1000/2022^a^	A/CS/1000/22	***G15***
22 Nov 2021	Poultry market	A/chicken/Fujian/S4075/2021	CK/FJ/S4075/21	***G1***
27 Nov 2021	Farm	A/duck/Hubei/S4513/2021	DK/HuB/S4513/21	G2
3 Dec 2021	Poultry market	A/duck/Zhejiang/S4867/2021	DK/ZJ/S4867/21	G3
7 Dec 2021	Farm	A/duck/Jiangxi/S40364/2021	DK/JX/S40364/21	G4
7 Dec 2021	Farm	A/duck/Jiangxi/S40560/2021	DK/JX/S40560/21	G5
8 Dec 2021	Poultry market	A/chicken/Guangdong/S4439/2021	CK/GD/S4439/21	***G1***
8 Dec 2021	Poultry market	A/duck/Guangdong/S4619/2021	DK/GD/S4619/21	***G1***
8 Dec 2021	Poultry market	A/chicken/Guangdong/S4451/2021	CK/GD/S4451/21	***G6***
7 Jan 2022	Poultry market	A/chicken/Hunan/SE204/2022	CK/HuN/SE204/22	***G7***
7 Jan 2022	Poultry market	A/goose/Hunan/SE235/2022	GS/HuN/SE235/22	***G8***
10 Jan 2022	Poultry market	A/chicken/Guangxi/S11685/2022	CK/GX/S11685/22	***G7***
21 Feb 2022	Poultry market	A/chicken/Guizhou/S1058/2022	CK/GZ/S1058/22	***G7***
1 Mar 2022	Poultry market	A/chicken/Fujian/S1186/2022	CK/FJ/S1186/22	***G1***
1 Mar 2022	Poultry market	A/chicken/Fujian/S1353/2022	CK/FJ/S1353/22	***G1***
3 Mar 2022	Farm	A/duck/Anhui/S1618/2022	DK/AH/S1618/22	G9
3 Mar 2022	Poultry market	A/duck/Anhui/S1780/2022	DK/AH/S1780/22	G10
14 Apr 2022	Poultry market	A/chicken/Jiangsu/S1009/2022	CK/JS/S1009/22	***G11***
24 Apr 2022	Poultry market	A/chicken/Ningxia/S1058/2022	CK/NX/S1058/22	***G1***
25 Apr 2022	Poultry market	A/chicken/Ningxia/S1230/2022	CK/NX/S1230/22	***G12***
27 Apr 2022	Poultry market	A/chicken/Ningxia/S1613/2022	CK/NX/S1613/22	***G13***
27Apr 2022	Poultry market	A/goose/Jiangsu/S1507/2022	GS/JS/S1507/22	***G11***
6 May 2022	Poultry market	A/duck/Zhejiang/S1761/2022	DK/ZJ/S1761/22	***G12***
6 May 2022	Poultry market	A/chicken/Zhejiang/S1784/2022	CK/ZJ/S1784/22	***G12***
10 May 2022	Poultry market	A/chicken/Guangdong/S1122/2022	CK/GD/S1122/22	***G7***
10 May 2022	Poultry market	A/chicken/Guangdong/S1292/2022	CK/GD/S1292/22	***G7***
10 May 2022	Poultry market	A/duck/Guangdong/S1323/2022	DK/GD/S1323/22	G14
18 May 2022	Poultry market	A/chicken/Hunan/S2076/2022	CK/HuN/S2076/22	***G7***
18 May 2022	Poultry market	A/chicken/Hunan/S2221/2022	CK/HuN/S2221/22	***G16***
18 May 2022	Poultry market	A/chicken/Hunan/S2283/2022	CK/HuN/S2283/22	***G16***
19 May 2022	Poultry market	A/chicken/Hunan/S2342/2022	CK/HuN/S2342/22	***G15***
19 May 2022	Poultry market	A/chicken/Henan/S1188/2022	CK/HeN/S1188/22	***G17***

^a^ The sequences of the human H3N8 viruses were downloaded from the GISAID EpiFlu Database.

^b^ Genotypes of viruses carrying the six internal genes of poultry H9N2 virus are shown in bold italics.

A. The time-scaled phylogenetic tree of the HA genes was inferred by using an asymmetric continuous-time Markov chain with Bayesian stochastic search variable selection implemented in BEAST (v1.10.4). The 31 viruses shown in blue were sequenced in this study, the two human viruses are shown in red, and the 26 representative viruses downloaded from the GISAID EpiFlu Database are shown in black. *, virus tested in guinea pigs; #, virus tested in chickens.

B. Genotype of the H3N8 viruses. The eight bars represent the eight gene segments, and the colour of the bar indicates the closest donor strain of the gene segment. The genotype for each viral strains is indicated together with, in parentheses, the number of corresponding viral isolates.

Based on their genomic differences, the 31 H3N8 viruses recovered from birds or their environments and two H3N8 viruses recovered from humans were categorised into 17 genotypes (G1 to G17) (Table 2). The 24 viruses bearing the group 1 HA and NA genes were mainly recovered from chicken, but also included the two viruses found in humans. The group 1 comprised 10 different genotypes (G1, G6–G8, G11–G13, and G15–G17) due to the diversity of their internal genes, although the six internal genes of these viruses were all highly related to the H9N2 viruses detected in domestic poultry in China (Figure 1, panel B; Supplementary Figure S2); the two H3N8 viruses—A/Henan/4–14CNIC/2022(H3N8) and A/Changsha/1000/2022(H3N8)—detected in humans belonged to genotypes G1 and G15, respectively (Figure 1, panel B). The rest of the seven H3N8 viruses were retrieved from ducks and their HA genes formed two groups (group 4 and group 5) composed of a total of seven genotypes (G2–G5, G9–G10, and G14), with all but one of their genes originating from wild bird viruses. These results suggest that H3N8 viruses from wild birds continue to spread in ducks in China, and some earlier strains (i.e. viruses bearing the HA of group 1) that may have been introduced in farmed ducks around August 2016 (according to the most recent common ancestor in phylogenetic analysis) have reassorted with H9N2 viruses in live poultry markets. The sequences of viruses causing the two human infections in 2022 bore evidence of these reassortments.

### Molecular characteristics of the H3N8 viruses

Several amino-acid residues are known to increase influenza virus binding to human-type receptor or to promote the replication and virulence of avian influenza viruses in mammals [14-18]. We analysed amino-acid substitutions with the FluSurver in GISAID and found that some of these residues, including 155T and 255G in HA, 309D, 431M, and 504V in PB2, 622G in PB1, 383D, 550L, and 639T in PA, 286A and 437T in NP, and 30D, 43M, and 215A in M1 are highly conserved and present in all of the 31 avian viruses we analysed and the two viruses found in humans reported by others, as also described in the Supplementary Table S2. Other residues, including 193N in HA, 292V, 598I/T, 627V, and 702R in PB2, 63I, 356R, and 409N in PA, and 42S in NS1 were also detected in some of these viruses, including the two involved in human infections. Moreover, in one of the viruses affecting humans (A/Henan/4–14CNIC/2022(H3N8)), 228S in HA and 627K in PB2 were also found (Table S2). In addition, 31N in M2, which is responsible for increased resistance to amantadine was detected in 23 of the H3N8 viruses detected in birds and the two strains detected in humans (Table S2). These results indicate that the H3N8 avian influenza viruses bear multiple residues that may favour their binding to human-type receptors and their replication in mammals.

### Replication and pathogenicity of H3N8 viruses in mice

**Figure 2:**
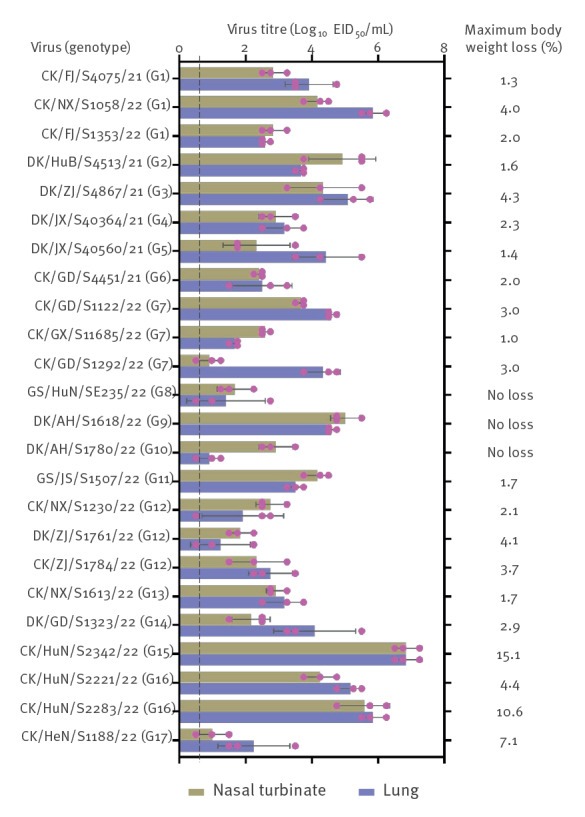
Replication and virulence in mice of avian H3N8 viruses isolated in China in September 2021−May 2022 (n = 24 strains)

To further investigate the replication and pathogenicity of H3N8 viruses in mammals, we selected 24 representative strains for mouse studies. We selected one virus each from the G2, G3, G4, G5, G6, G8, G9, G10, G11, G13, G14, G15, and G17 genotypes. Since the viruses in the G1, G7, G12, and G16 genotypes were isolated from different markets, we selected three viruses each from the G1, G7, and G12 genotypes, and two from the G16 genotype. It is worth pointing out that the H3N8 viruses, which had infected humans were not tested in this study as we did not have these strains. Groups of eight 6-week-old female BALB/c mice were inoculated intranasally with 10^6^ EID_50_ of test virus. Three mice in each group were euthanised on day 3 p.i. and their organs were collected for virus titration in eggs; the remaining mice were observed for 2 weeks. We found that all 24 viruses replicated in the nasal turbinate and lungs of mice, but the viral titre levels varied among the different strains (Figure 2). No virus was detected in the spleens, kidneys, or brains of the mice inoculated with any virus. Twenty-one of the 24 viruses caused 1.3–15.1% body weight loss in mice, but all of the mice survived during the two-week observation period. These results demonstrate that the H3N8 viruses replicate efficiently in mice, but are not lethal.

Virus titres in organs of mice inoculated intranasally with 10^6^ EID_50_ of different H3N8 viruses. Data shown are means ± standard deviations. The dashed lines indicate the lower limit of detection. The maximum body weight loss was calculated as the difference between the lowest values and the initial values of the infected mice during the 14-day observation period.

### Receptor-binding specificity of the H3N8 viruses

Binding to the human-type receptor is a prerequisite for an influenza virus to infect and transmit effectively in humans. To evaluate the receptor-binding properties of the H3N8 viruses, nine strains that replicated efficiently in both the nasal turbinate and lungs of mice were tested by using a solid-phase binding assay as described previously [13]. We found that all nine viruses bound to α-2, 3-linked sialic acid (avian-type receptor) with high affinity; however, their affinity for α-2, 6-linked sialic acid (human-type receptor) varied among the strains, and the affinity of DK/HuB/S4513/21 for the human-type receptor was the lowest among the nine strains, as illustrated by the graph of Supplementary Figure S3. Of note, the DK/HuB/S4513/21 has a 193S in its HA, whereas the other eight strains have a 193N in their HAs (Supplementary Table S2). Whether this difference leads to differences in receptor binding of these strains remains to be investigated.

### Replication and transmission of H3N8 viruses in guinea pigs

Effective human-to-human transmission is a prerequisite for pandemic influenza viruses and is therefore one of the most undesirable features of emerging influenza viruses. Although we found that some of the emerging H3N8 viruses could bind to human-type receptors and replicate efficiently in mice, it remains to be investigated whether they can transmit from person to person. To look into possible transmission in mammals, six H3N8 viral strains that replicated efficiently in the organs of mice and bound to α-2,6-linked sialic acids with relatively high affinity were selected for respiratory droplet transmission studies in guinea pigs.

**Figure 3:**
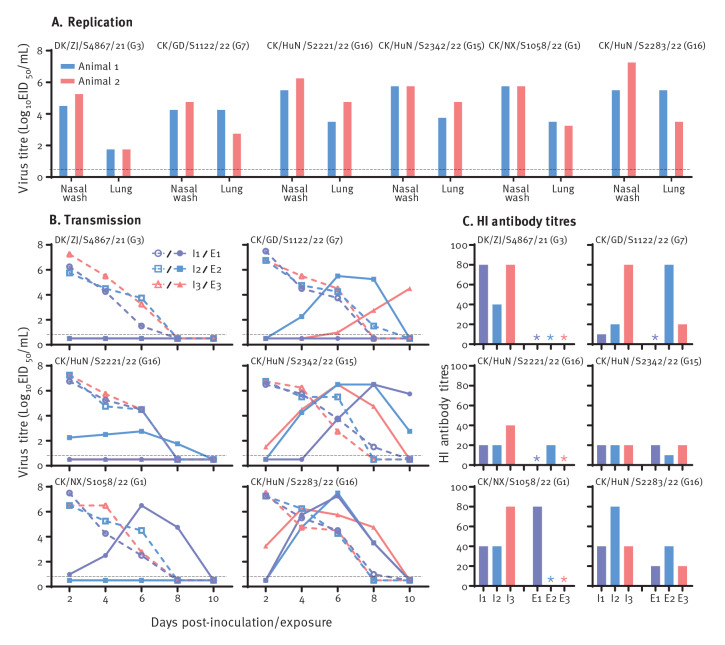
Replication and respiratory droplet transmission in guinea pigs of H3N8 viruses isolated in China in September 2021−May 2022 (n = 6 strains)

We found that all six viruses replicated well in the upper respiratory tract of guinea pigs, with viral titres ranging from 4.3 to 7.3 log_10_ EID_50_ in the nasal washes on day 3 p.i., and in the lungs, with viral titres ranging from 1.8 to 5.5 log_10_ EID_50_ (Figure 3, panel A). In the transmission study, virus was detected on days 2, 4, and 6 p.i. in the nasal washes of the animals that were inoculated with any of the six strains (Figure 3, panel B). In the exposed animals, virus was not detected in the nasal washes of the animals exposed to DK/ZJ/S4867/2021, but was detected in one animal exposed to CK/HuN/S2221/22 or CK/NX/S1058/22, two animals exposed to CK/GD/S1122/22, and all three animals exposed to CK/HuN/S2342/22 or CK/HuN/S2283/22 (Figure 3, panel B). Seroconversion occurred in all inoculated animals and virus-shedding-positive exposed animals (Figure 3, panel C). The HI antibody levels of the CK/HuN/S2221/22- and CK/HuN/S2342/22-infected animals were relatively low, possibly because these two viruses may have relatively poor immunogenicity. These results indicate that five of the six test viruses were transmissible in guinea pigs via respiratory droplets. Of note, all of these transmissible viruses carried the internal genes of H9N2 viruses.

E: exposed; EID_50_: 50% egg infectious dose; HI: haemagglutinin inhibition; I: inoculated.

A. Replication of H3N8 virus in guinea pigs.

B. Respiratory droplet transmission of H3N8 viruses in guinea pigs.

C. HI antibody titres of H3N8 virus-inoculated or H3N8 virus-exposed guinea pigs. Each colour line or bar represents the virus titre from an individual animal.

The name of the virus tested is at the top of each graph, with the genotype indicated in parentheses. The horizontal dashed black lines in panels A and B indicate the lower limit of detection. The asterisks in panels C indicate the animals that did not seroconvert.

### Replication and virulence of H3N8 viruses in chickens

**Figure 4:**
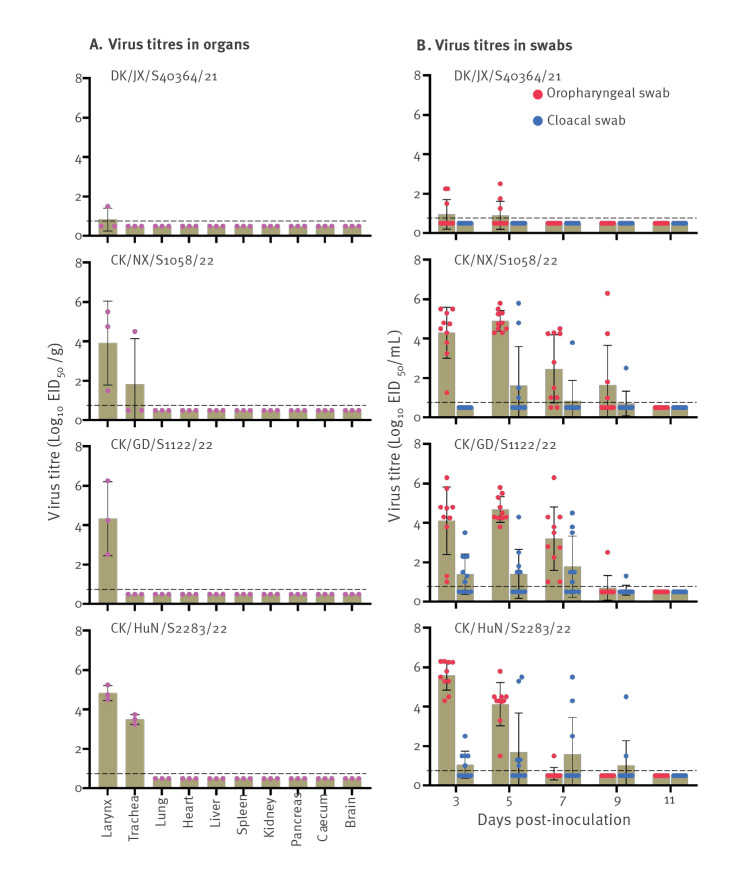
Replication and virulence, in chickens, of H3N8 viruses isolated in China in September 2021−May 2022 (n = 4 strains)

The H3N8 viruses in the present study were all isolated from apparently healthy poultry during our surveillance; however, Yang et al. and Wan et al. reported the isolation of H3N8 viruses from the organs, including lungs, of diseased chickens [19,20]. To investigate whether H3N8 virus causes disease in chickens, we selected and tested the following four viruses: a duck virus (i.e. DK/JX/S40364/2021; G4) that does not bear the internal genes of H9N2 virus and three chicken viruses (i.e. CK/NX/S1058/2022 (G1), CK/GD/S1122/2022 (G7), and CK/HuN/S2283/22 (G16)) that bear the internal genes of H9N2 viruses. Virus replication in organs was examined on day 3 p.i. and virus shedding was monitored for 11 days. We found that all four viruses could be detected in the larynx of chickens, and two of them could also be detected in the trachea of chickens, with titres varying among strains. Of note, the viruses were not detected in any other organs tested (Figure 4, panel A). Only three of the 10 chickens inoculated with DK/JX/S40364/2021 shed virus through the oropharynx on days 3 p.i. and 5 p.i., whereas all chickens inoculated with any of the other three strains bearing the H9N2 virus internal genes shed virus through the oropharynx, and some also shed virus through the cloaca (Figure 4, panel B). Although virus shedding by some birds could be detected for up to 9 days after inoculation, all of the chickens remained healthy and survived the 2-week observation period. These results indicate that the H3N8 viruses mainly replicate in the upper respiratory tract of chickens and do not cause disease in these birds. Of note, the increased replication in chickens of the H3N8 viruses bearing the H9N2 internal genes may have partially contributed to the wider detection of the H3N8 viruses in live poultry markets in 2022.

A. Viral titres in the organs of chickens inoculated with the indicated viruses.

B. Viral shedding in swabs of chickens inoculated with the indicated viruses.

The name of the virus tested is at the top of each graph. Data shown are means ± standard deviations. The dashed lines indicate the lower limit of detection.

## Discussion

The H3N8 influenza viruses have been detected in multiple animal species in nature, including different wild birds, domestic poultry, horses, donkeys, dogs, and harbour seals [21-25]. In this study, we detected different genotypes of H3N8 viruses in farmed ducks and different avian species in live poultry markets, and found that some viruses detected in the live poultry markets have acquired the six internal genes of H9N2 viruses. 

Previous studies have shown that the H9N2 viruses are widely detected in live poultry markets in China [16], and chickens vaccinated with the H9N2 vaccines can still be reinfected by H9N2 viruses in these markets [26]. Such a situation provides an opportunity for the H9N2 viruses to reassort with other subtype viruses in the markets, as evidenced by the emergence of H7N9 and H10N8 viruses in 2013, H10N3 viruses in 2020, and the 24 H3N8 viruses detected in this study all bearing six internal genes derived from H9N2 viruses [9,10,27].

Moreover, in our investigations, H3N8 viruses bearing the H9N2 internal genes replicated well in the upper respiratory tract of chickens and were efficiently shed by chickens through both the oropharynx and cloaca. These viruses were also transmissible between guinea pigs via respiratory droplets. Importantly, such H3N8 viruses caused two human infection cases in China [11,12]. This suggests that viruses with this gene constellation may be able to circulate in chickens and continue to pose a threat to human health.

An emerging influenza virus that can replicate in humans may acquire key amino-acid substitutions to adapt to humans, and some human-adaptation mutations also play a key role in facilitating the spread of the virus from human to human. One of the best-known substitutions is the E to K substitution at position 627 (E627K) in PB2, which can dramatically increase the virulence and promote the transmissibility of avian influenza virus in mammals. A previous study indicated that over 78% of the H7N9 avian influenza viruses acquired the 627K substitution in PB2 after replication in humans [28]. Liang et al. found that the low polymerase activity attributed to the viral PA protein is the intrinsic driving force behind the emergence of PB2 627K during H7N9 virus replication in mammals [29]. Several studies have reported that the E627V substitution in PB2 increases the replication and lethality of different avian influenza viruses in mice [30,31]. Zhang et al. found that the H3N2 avian influenza virus easily acquires the 226R/L or 228S substitution in HA during its replication in ferrets, and that either of these substitutions can appreciably increase its affinity for the human-like receptor, allowing the virus to be readily transmitted in ferrets via respiratory droplets. Further analysis indicated that the 228S substitution in HA is important and necessary for the H3N2 virus to be a pandemic strain [13]. Both 627K in PB2 and 228S in HA were detected in the A/Henan/4–14CNIC/2022 strain (Table S2), that was isolated from an infected child’s clinical sample, indicating that the H3N8 virus could easily obtain these key substitutions after a single round of replication in humans. In addition, we found that three avian H3N8 viruses (i.e. CK/HuN/S2342/22 (G15), CK/HuN/S2221/22 (G16), and CK/HuN/S2283/22 (G16)) have the E627V substitution in their PB2 (Table S2), which may contribute to their efficient replication in mice (Figure 2). Therefore, we must carefully monitor H3N8 virus infection in humans and evaluate the human-to-human transmission potential of the viruses isolated from humans in order to be prepared for future influenza pandemics.

One limitation of this study is that the two H3N8 viral strains that had infected humans were neither assessed in the receptor-binding assays nor in the animal investigations as we did not have these strains.

### Conclusion

Our study detected multiple genotypes of H3N8 viruses in farmed ducks and different birds in the live poultry markets in China, and found that the proportion of H3N8 viruses bearing the six internal genes of H9N2 virus appeared high in 2022 in multiple live poultry markets and caused two human infections. Some H3N8 viruses bind to both avian-type and human-type receptors, and are transmissible in guinea pigs via respiratory droplets, indicating clear pandemic potential. The low pathogenicity of the H3N8 avian influenza virus in poultry has led to its control being a low priority, which allows the virus to continue to circulate and pose a threat to human health. Carefully monitoring of the H3N8 virus in humans is important to strengthen early warning systems and pandemic preparedness.

## Supplementary Data

3464000 Supplementary Material

## References

[1] Wille M Holmes EC . The Ecology and Evolution of Influenza Viruses. Cold Spring Harb Perspect Med. 2020;10(7):a038489. 10.1101/cshperspect.a038489 31871237 PMC7328453

[2] Lee DH Criado MF Swayne DE . Pathobiological Origins and Evolutionary History of Highly Pathogenic Avian Influenza Viruses. Cold Spring Harb Perspect Med. 2021;11(2):a038679. 10.1101/cshperspect.a038679 31964650 PMC7849344

[3] Wang D Zhu W Yang L Shu Y . The Epidemiology, Virology, and Pathogenicity of Human Infections with Avian Influenza Viruses. Cold Spring Harb Perspect Med. 2021;11(4):a038620. 10.1101/cshperspect.a038620 31964651 PMC8015695

[4] Cui P Zeng X Li X Li Y Shi J Zhao C Genetic and biological characteristics of the globally circulating H5N8 avian influenza viruses and the protective efficacy offered by the poultry vaccine currently used in China. Sci China Life Sci. 2022;65(4):795-808. 10.1007/s11427-021-2025-y 34757542

[5] Cui P Shi J Wang C Zhang Y Xing X Kong H Global dissemination of H5N1 influenza viruses bearing the clade 2.3.4.4b HA gene and biologic analysis of the ones detected in China. Emerg Microbes Infect. 2022;11(1):1693-704. 10.1080/22221751.2022.2088407 35699072 PMC9246030

[6] Gu W Shi J Cui P Yan C Zhang Y Wang C Novel H5N6 reassortants bearing the clade 2.3.4.4b HA gene of H5N8 virus have been detected in poultry and caused multiple human infections in China. Emerg Microbes Infect. 2022;11(1):1174-85. 10.1080/22221751.2022.2063076 35380505 PMC9126593

[7] Zeng X Tian G Shi J Deng G Li C Chen H . Vaccination of poultry successfully eliminated human infection with H7N9 virus in China. Sci China Life Sci. 2018;61(12):1465-73. 10.1007/s11427-018-9420-1 30414008

[8] Yuan J Zhang L Kan X Jiang L Yang J Guo Z Origin and molecular characteristics of a novel 2013 avian influenza A(H6N1) virus causing human infection in Taiwan. Clin Infect Dis. 2013;57(9):1367-8. 10.1093/cid/cit479 23881153

[9] Qi X Qiu H Hao S Zhu F Huang Y Xu K Human Infection with an Avian-Origin Influenza A (H10N3) Virus. N Engl J Med. 2022;386(11):1087-8. 10.1056/NEJMc2112416 35294820

[10] Chen H Yuan H Gao R Zhang J Wang D Xiong Y Clinical and epidemiological characteristics of a fatal case of avian influenza A H10N8 virus infection: a descriptive study. Lancet. 2014;383(9918):714-21. 10.1016/S0140-6736(14)60111-2 24507376

[11] Cheng D Dong Y Wen S Shi C . A child with acute respiratory distress syndrome caused by avian influenza H3N8 virus. J Infect. 2022;85(2):174-211. 10.1016/j.jinf.2022.05.007 35577072

[12] Tan X Yan X Liu Y Wu Y Liu J Mu M A case of human infection by H3N8 influenza virus. Emerg Microbes Infect. 2022;11(1):2214-7. 10.1080/22221751.2022.2117097 36000153 PMC9542523

[13] Zhang Y Zhao C Hou Y Chen Y Meng F Zhuang Y Pandemic threat posed by H3N2 avian influenza virus. Sci China Life Sci. 2021;64(11):1984-7. 10.1007/s11427-021-1916-4 33765225

[14] Ma S Zhang B Shi J Yin X Wang G Cui P Amino Acid Mutations A286V and T437M in the Nucleoprotein Attenuate H7N9 Viruses in Mice. J Virol. 2020;94(2):e01530-19. 10.1128/JVI.01530-19 31666373 PMC6955278

[15] Kong X Guan L Shi J Kong H Zhang Y Zeng X A single-amino-acid mutation at position 225 in hemagglutinin attenuates H5N6 influenza virus in mice. Emerg Microbes Infect. 2021;10(1):2052-61. 10.1080/22221751.2021.1997340 34686117 PMC8583753

[16] Li X Shi J Guo J Deng G Zhang Q Wang J Genetics, receptor binding property, and transmissibility in mammals of naturally isolated H9N2 Avian Influenza viruses. PLoS Pathog. 2014;10(11):e1004508. 10.1371/journal.ppat.1004508 25411973 PMC4239090

[17] Feng X Wang Z Shi J Deng G Kong H Tao S Glycine at Position 622 in PB1 Contributes to the Virulence of H5N1 Avian Influenza Virus in Mice. J Virol. 2016;90(4):1872-9. 10.1128/JVI.02387-15 26656683 PMC4733975

[18] Fan S Deng G Song J Tian G Suo Y Jiang Y Two amino acid residues in the matrix protein M1 contribute to the virulence difference of H5N1 avian influenza viruses in mice. Virology. 2009;384(1):28-32. 10.1016/j.virol.2008.11.044 19117585

[19] Yang R Sun H Gao F Luo K Huang Z Tong Q Human infection of avian influenza A H3N8 virus and the viral origins: a descriptive study. Lancet Microbe. 2022;3(11):e824-34. 10.1016/S2666-5247(22)00192-6 36115379

[20] Wan Z Jiang W Gong J Zhao Z Tang T Li Y Emergence of chicken infection with novel reassortant H3N8 avian influenza viruses genetically close to human H3N8 isolate, China. Emerg Microbes Infect. 2022;11(1):2553-5. 10.1080/22221751.2022.2128437 36150006 PMC9621203

[21] Deng G Tan D Shi J Cui P Jiang Y Liu L Complex reassortment of multiple subtypes of avian influenza viruses in domestic ducks at the Dongting Lake Region of China. J Virol. 2013;87(17):9452-62. 10.1128/JVI.00776-13 23804642 PMC3754128

[22] Yang H Xiao Y Meng F Sun F Chen M Cheng Z Emergence of H3N8 equine influenza virus in donkeys in China in 2017. Vet Microbiol. 2018;214:1-6. 10.1016/j.vetmic.2017.11.033 29408020

[23] Anthony SJ St Leger JA Pugliares K Ip HS Chan JM Carpenter ZW Emergence of fatal avian influenza in New England harbor seals. mBio. 2012;3(4):e00166-12. 10.1128/mBio.00166-12 22851656 PMC3419516

[24] Li Y Li P Xi J Yang J Wu H Zhang Y Wild bird-origin H3N8 avian influenza virus exhibit well adaptation in mammalian host. J Infect. 2022;84(4):579-613. 10.1016/j.jinf.2021.12.014 34953909

[25] Crawford PC Dubovi EJ Castleman WL Stephenson I Gibbs EP Chen L Transmission of equine influenza virus to dogs. Science. 2005;310(5747):482-5. 10.1126/science.1117950 16186182

[26] Li M Yin X Guan L Zhang X Deng G Li T Insights from avian influenza surveillance of chickens and ducks before and after exposure to live poultry markets. Sci China Life Sci. 2019;62(6):854-7. 10.1007/s11427-019-9522-7 30977013

[27] Zhang Q Shi J Deng G Guo J Zeng X He X H7N9 influenza viruses are transmissible in ferrets by respiratory droplet. Science. 2013;341(6144):410-4. 10.1126/science.1240532 23868922

[28] Shi J Deng G Kong H Gu C Ma S Yin X H7N9 virulent mutants detected in chickens in China pose an increased threat to humans. Cell Res. 2017;27(12):1409-21. 10.1038/cr.2017.129 29151586 PMC5717404

[29] Liang L Jiang L Li J Zhao Q Wang J He X Low Polymerase Activity Attributed to PA Drives the Acquisition of the PB2 E627K Mutation of H7N9 Avian Influenza Virus in Mammals. mBio. 2019;10(3):e01162-19. 10.1128/mBio.01162-19 31213560 PMC6581862

[30] Liu K Ding P Pei Y Gao R Han W Zheng H Emergence of a novel reassortant avian influenza virus (H10N3) in Eastern China with high pathogenicity and respiratory droplet transmissibility to mammals. Sci China Life Sci. 2022;65(5):1024-35. 10.1007/s11427-020-1981-5 34542812

[31] Taft AS Ozawa M Fitch A Depasse JV Halfmann PJ Hill-Batorski L Identification of mammalian-adapting mutations in the polymerase complex of an avian H5N1 influenza virus. Nat Commun. 2015;6(1):7491. 10.1038/ncomms8491 26082035 PMC4557292

