# Supplementary material for: Analysis of avian influenza A (H3N8) viruses in poultry and their zoonotic potential, China, September 2021 to May 2022
Source: Euro Surveill. 2023 Oct 12;28(41):2200871. doi: 10.2807/1560-7917.ES.2023.28.41.2200871 (PMC10571489; doi:10.2807/1560-7917.ES.2023.28.41.2200871)
Supplement: Supplementary Material [file 2200871_SupplementaryMaterial.pdf]

Supplementary Material

This supplementary material is hosted by Eurosurveillance as supporting information alongside the article 'Analysis of avian influenza A (H3N8) viruses in poultry and their zoonotic potential, China, September 2021 to May 2022', on behalf of the authors, who remain responsible for the accuracy and appropriateness of the content. The same standards for ethics, copyright, attributions and permissions as for the article apply. Supplements are not edited by Eurosurveillance and the journal is not responsible for the maintenance of any links or email addresses provided therein.

Supplementary Figure S1. Schematic presentation of the transmission cages.

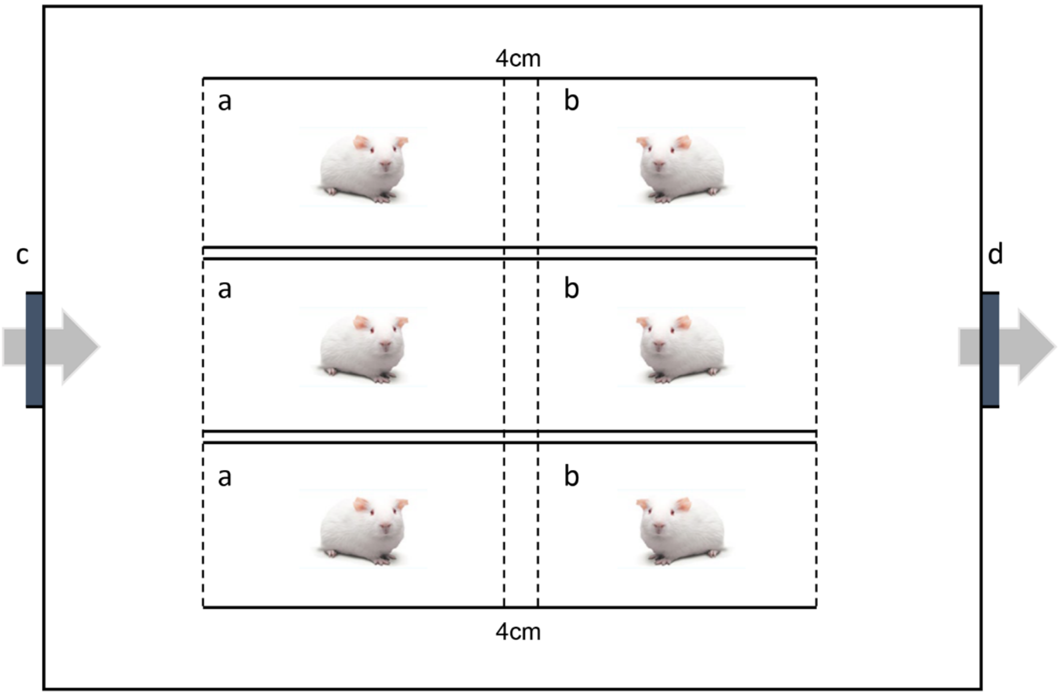

The transmission cages were specifically designed to allow transmission experiments to be conducted in negatively pressurized isolators (165 cm x 120 cm x 75 cm) in the ABSL2+ facility. The guinea pigs were housed in a clear stainless cage (18 cm x 54 cm x 35 cm), which was divided into 2 smaller cages (18 cm x 25 cm x 35 cm) by a double-layered net divider (the neighboring cages were 4 cm apart). Twenty-four hours after the three inoculated guinea pigs were placed into the cages (a), three naïve guinea pigs were placed in the adjacent cages (b). Negative pressure within the isolator cage was used to direct a modest (0.1 m/sec) flow of HEPA-filtered air (c) from the inoculated to the naïve animals. The outlet airflow (d) was HEPA-filtered to prevent the continuous circulation of infectious influenza virus particles.

Supplementary Figure S2. Phylogenetic analysis of the NA and six internal genes of the H3N8 viruses.

Phylogenetic analysis was performed by using the MEGA 7.0.14 software package, implementing the neighbor-joining method. The tree topology was evaluated by 1,000 bootstrap analyses, and 95% sequence identity cutoffs were used to categorize each gene segment in the phylogenetic trees. (Figure S2A-2F) The phylogenetic trees of the NA, PB2, PB1, PA, NP, and M genes were rooted to A/duck/Ukraine/1/1963 (H3N8). (Figure S2G) The phylogenetic tree of the NS gene was rooted in A/mallard/Alberta/11527/2005 (H3N8). The viruses sequenced in this study are shown in blue; the human H3N8 viruses are shown in red.

Figure S2A (NA)

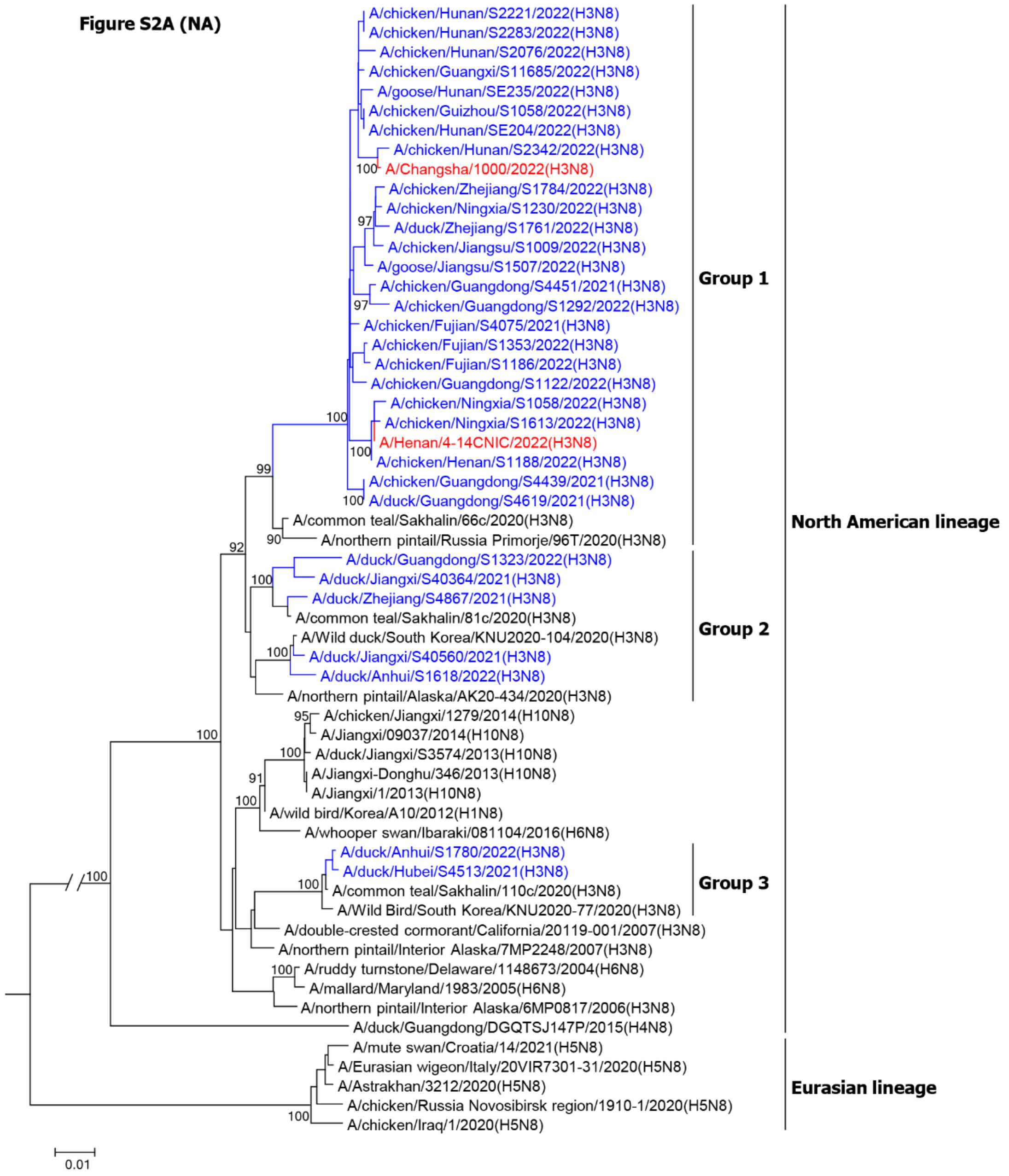

Figure S2B (PB2)

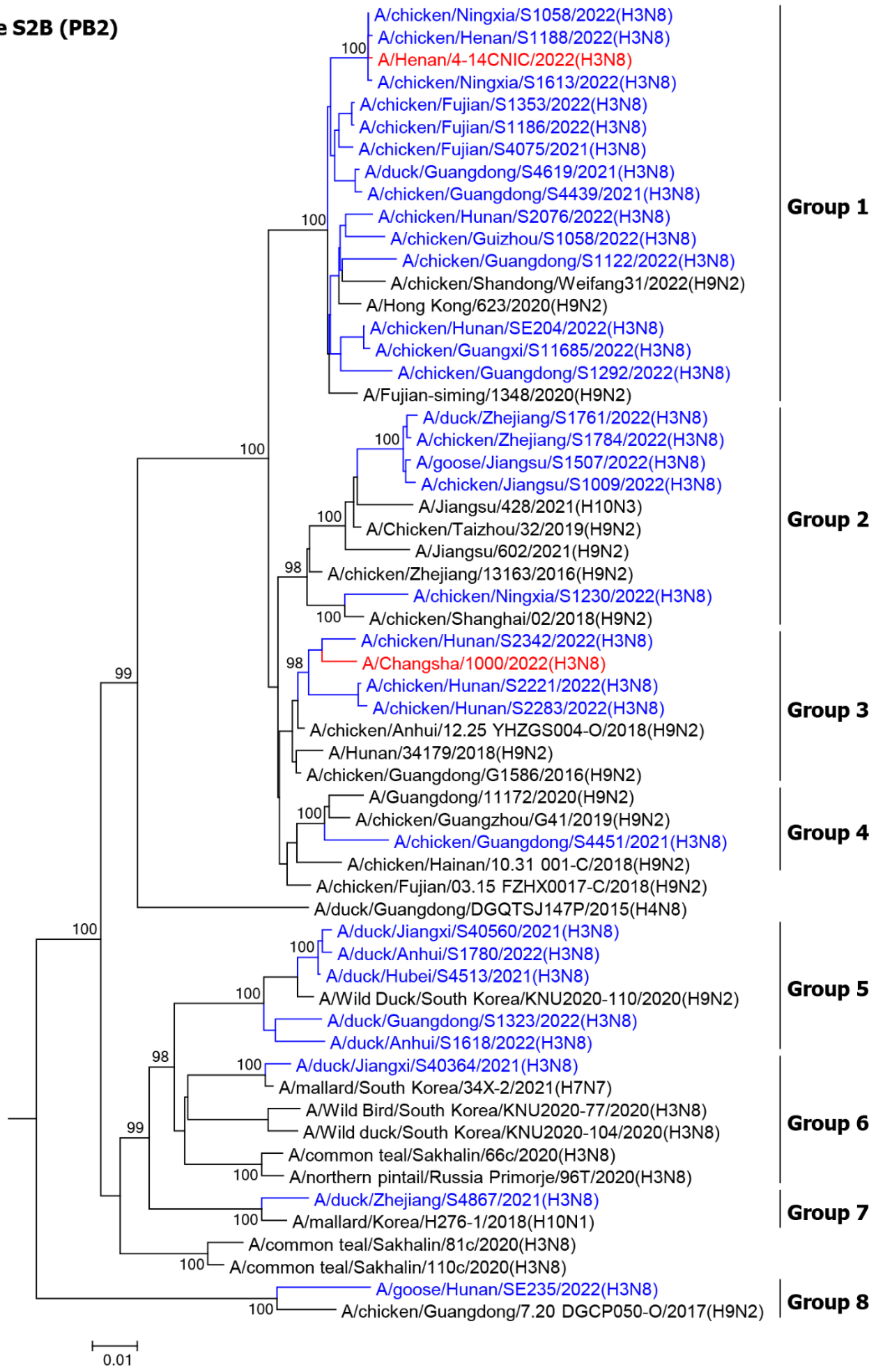

Figure S2C (PB1)

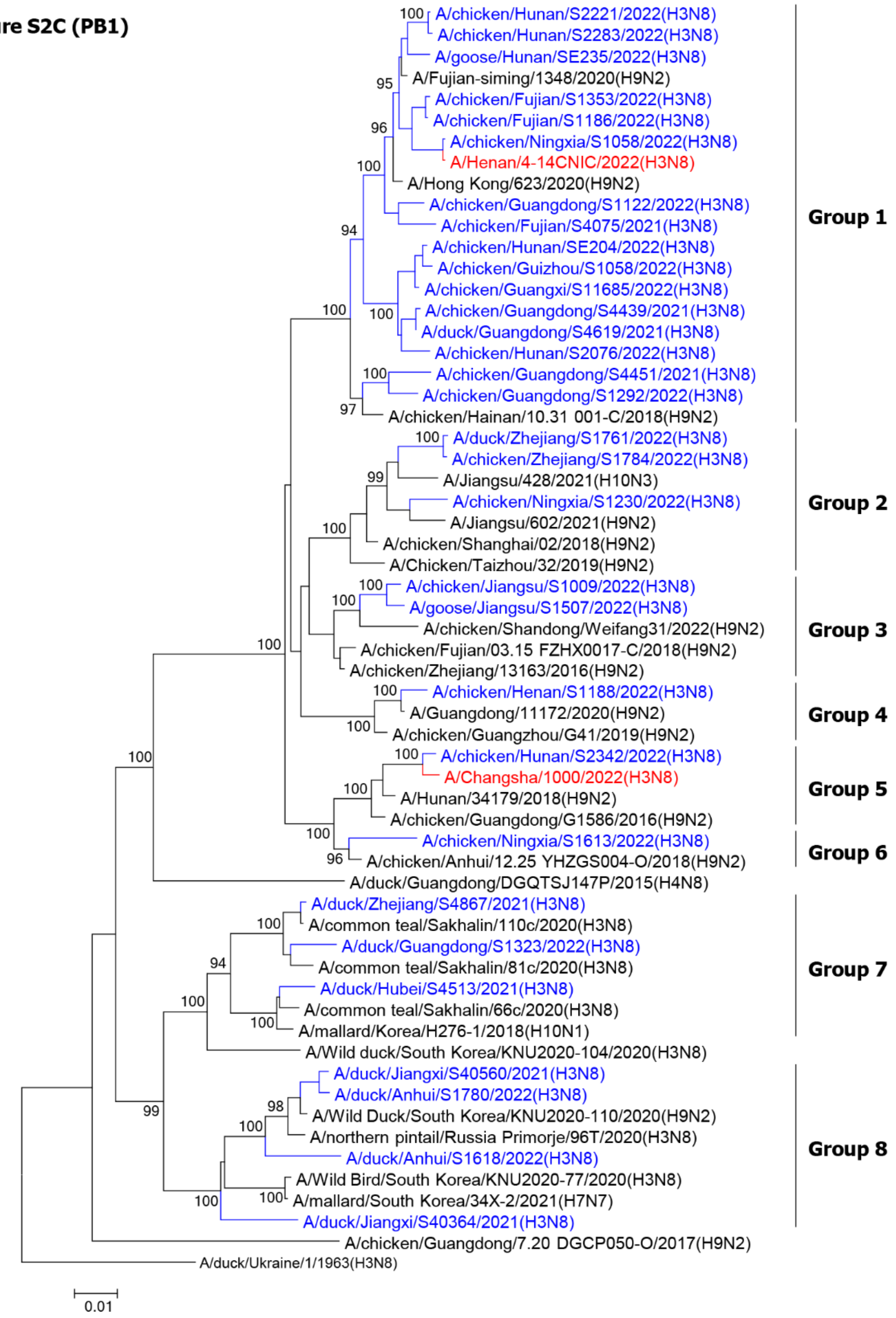

Figure S2D (PA)

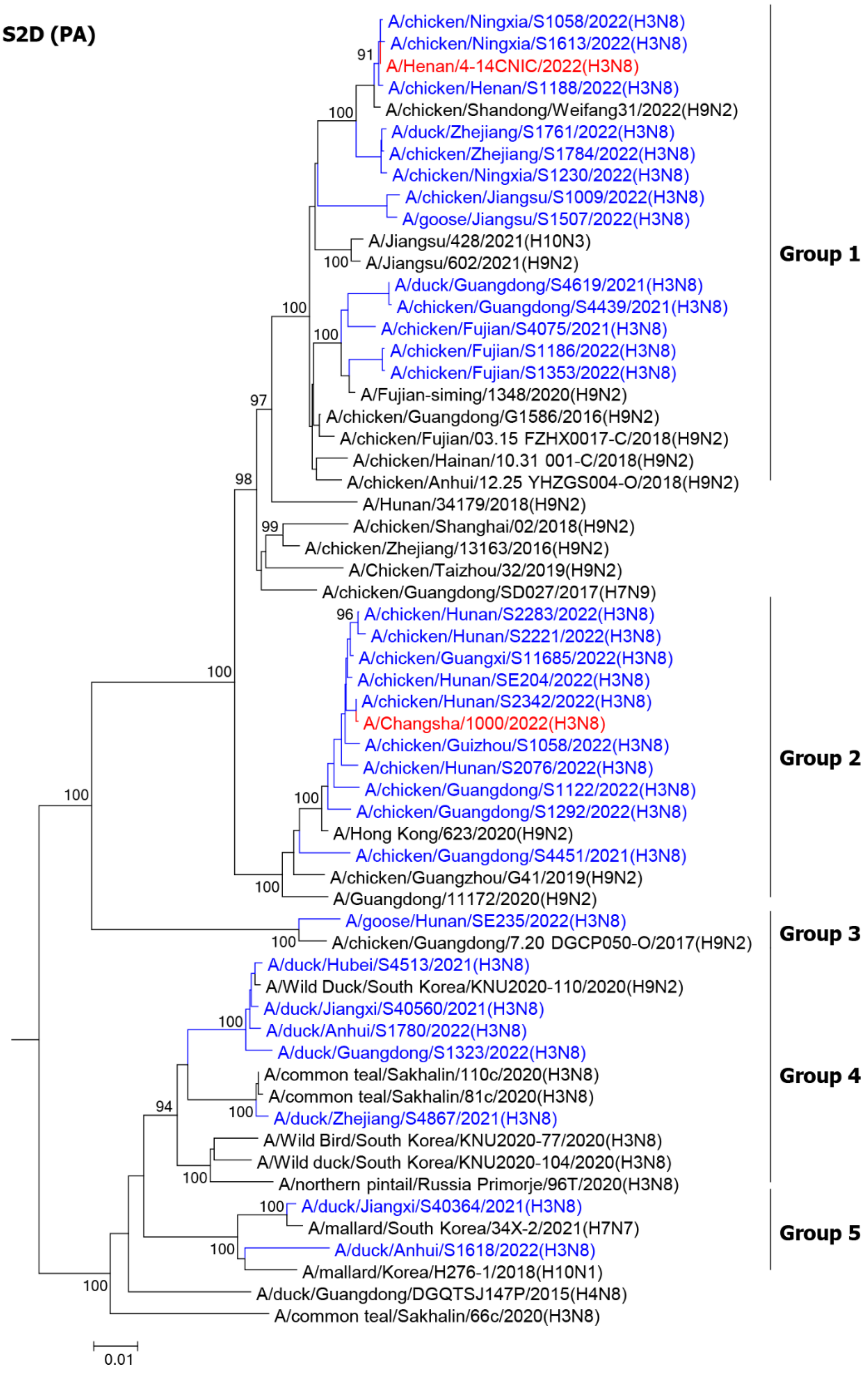

Figure S2E (NP)

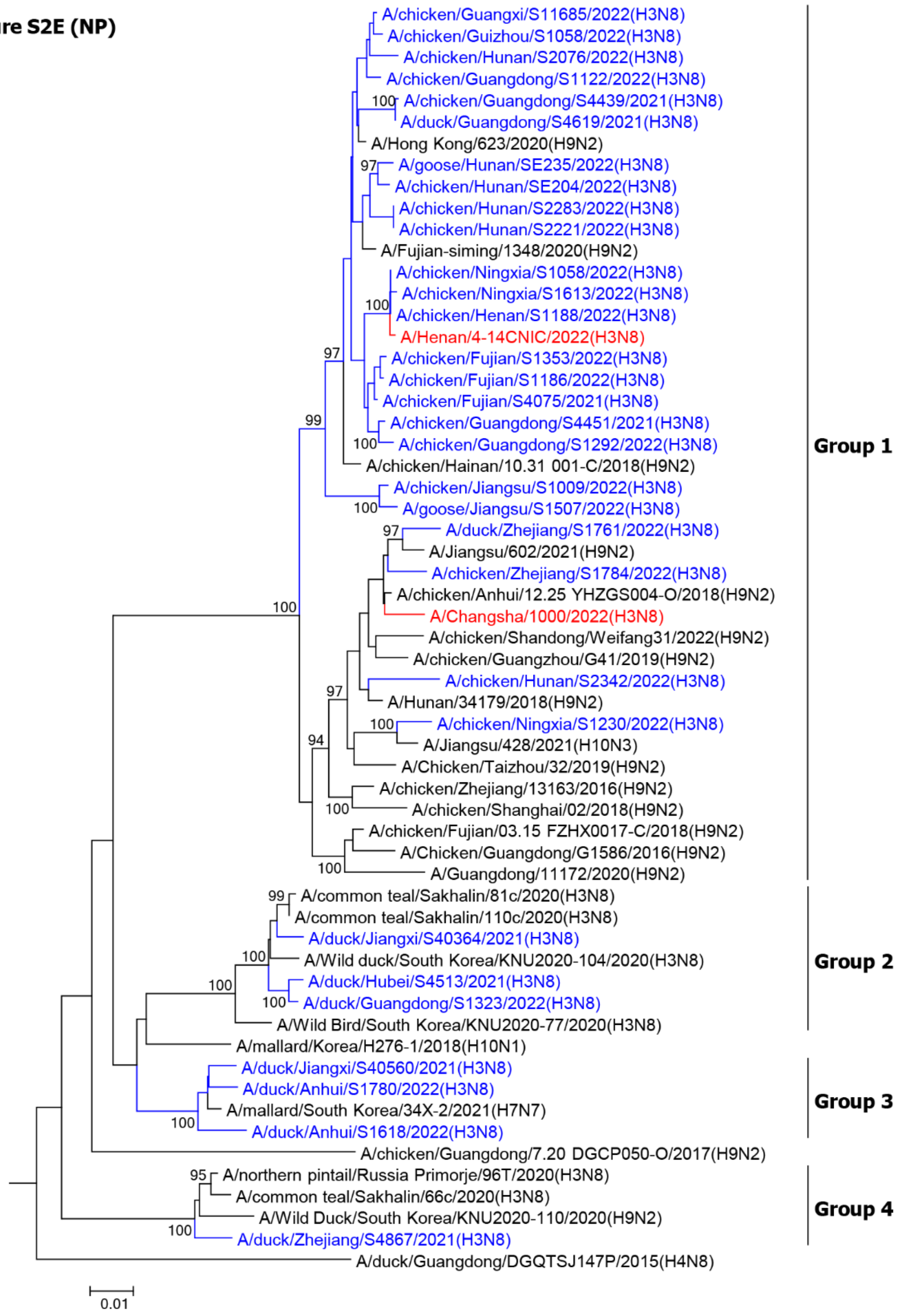

Figure S2F (M)

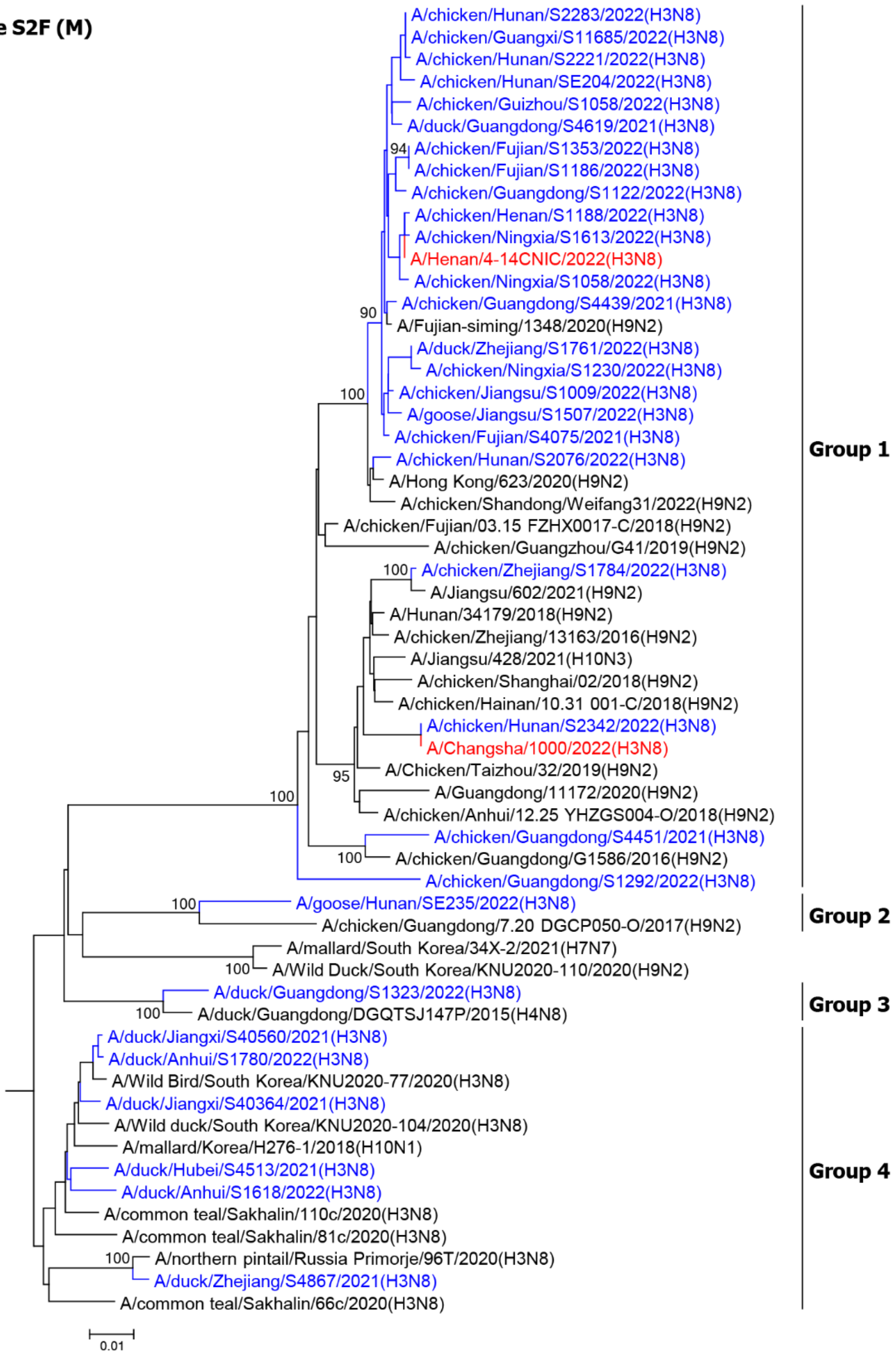
A/chicken/Hunan/S2283/2022(H3N8)
A/chicken/Guangxi/S11685/2022(H3N8)
A/chicken/Hunan/S2221/2022(H3N8)
A/chicken/Hunan/SE204/2022(H3N8)
A/chicken/Guizhou/S1058/2022(H3N8)
A/duck/Guangdong/S4619/2021(H3N8)
94
A/chicken/Fujian/S1353/2022(H3N8)
A/chicken/Fujian/S1186/2022(H3N8)
A/chicken/Guangdong/S1122/2022(H3N8)
A/chicken/Henan/S1188/2022(H3N8)
A/chicken/Ningxia/S1613/2022(H3N8)
A/Henan/4-14CNIC/2022(H3N8)
A/chicken/Ningxia/S1058/2022(H3N8)
90
A/chicken/Guangdong/S4439/2021(H3N8)
A/Fujian-siming/1348/2020(H9N2)
A/duck/Zhejiang/S1761/2022(H3N8)
A/chicken/Ningxia/S1230/2022(H3N8)
100
A/chicken/Jiangsu/S1009/2022(H3N8)
A/goose/Jiangsu/S1507/2022(H3N8)
A/chicken/Fujian/S4075/2021(H3N8)
A/chicken/Hunan/S2076/2022(H3N8)
A/Hong Kong/623/2020(H9N2)
A/chicken/Shandong/Weifang31/2022(H9N2)
A/chicken/Fujian/03.15 FZHX0017-C/2018(H9N2)
A/chicken/Guangzhou/G41/2019(H9N2)
100
A/chicken/Zhejiang/S1784/2022(H3N8)
A/Jiangsu/602/2021(H9N2)
A/Hunan/34179/2018(H9N2)
A/chicken/Zhejiang/13163/2016(H9N2)
A/Jiangsu/428/2021(H10N3)
A/chicken/Shanghai/02/2018(H9N2)
A/chicken/Hainan/10.31 001-C/2018(H9N2)
A/chicken/Hunan/S2342/2022(H3N8)
A/Changsha/1000/2022(H3N8)
95
A/Chicken/Taizhou/32/2019(H9N2)
A/Guangdong/11172/2020(H9N2)
100
A/chicken/Anhui/12.25 YHZGS004-O/2018(H9N2)
A/chicken/Guangdong/S4451/2021(H3N8)
100
A/chicken/Guangdong/G1586/2016(H9N2)
A/chicken/Guangdong/S1292/2022(H3N8)
100
A/goose/Hunan/SE235/2022(H3N8)
A/chicken/Guangdong/7.20 DGCP050-O/2017(H9N2)
A/mallard/South Korea/34X-2/2021(H7N7)
100
A/Wild Duck/South Korea/KNU2020-110/2020(H9N2)
A/duck/Guangdong/S1323/2022(H3N8)
100
A/duck/Guangdong/DGQTSJ147P/2015(H4N8)
A/duck/Jiangxi/S40560/2021(H3N8)
A/duck/Anhui/S1780/2022(H3N8)
A/Wild Bird/South Korea/KNU2020-77/2020(H3N8)
A/duck/Jiangxi/S40364/2021(H3N8)
A/Wild duck/South Korea/KNU2020-104/2020(H3N8)
A/mallard/Korea/H276-1/2018(H10N1)
A/duck/Hubei/S4513/2021(H3N8)
A/duck/Anhui/S1618/2022(H3N8)
A/common teal/Sakhalin/110c/2020(H3N8)
A/common teal/Sakhalin/81c/2020(H3N8)
100
A/northern pintail/Russia Primorje/96T/2020(H3N8)
A/duck/Zhejiang/S4867/2021(H3N8)
A/common teal/Sakhalin/66c/2020(H3N8)
0.01
Group 1
Group 2
Group 3
Group 4

Figure S2G (NS)

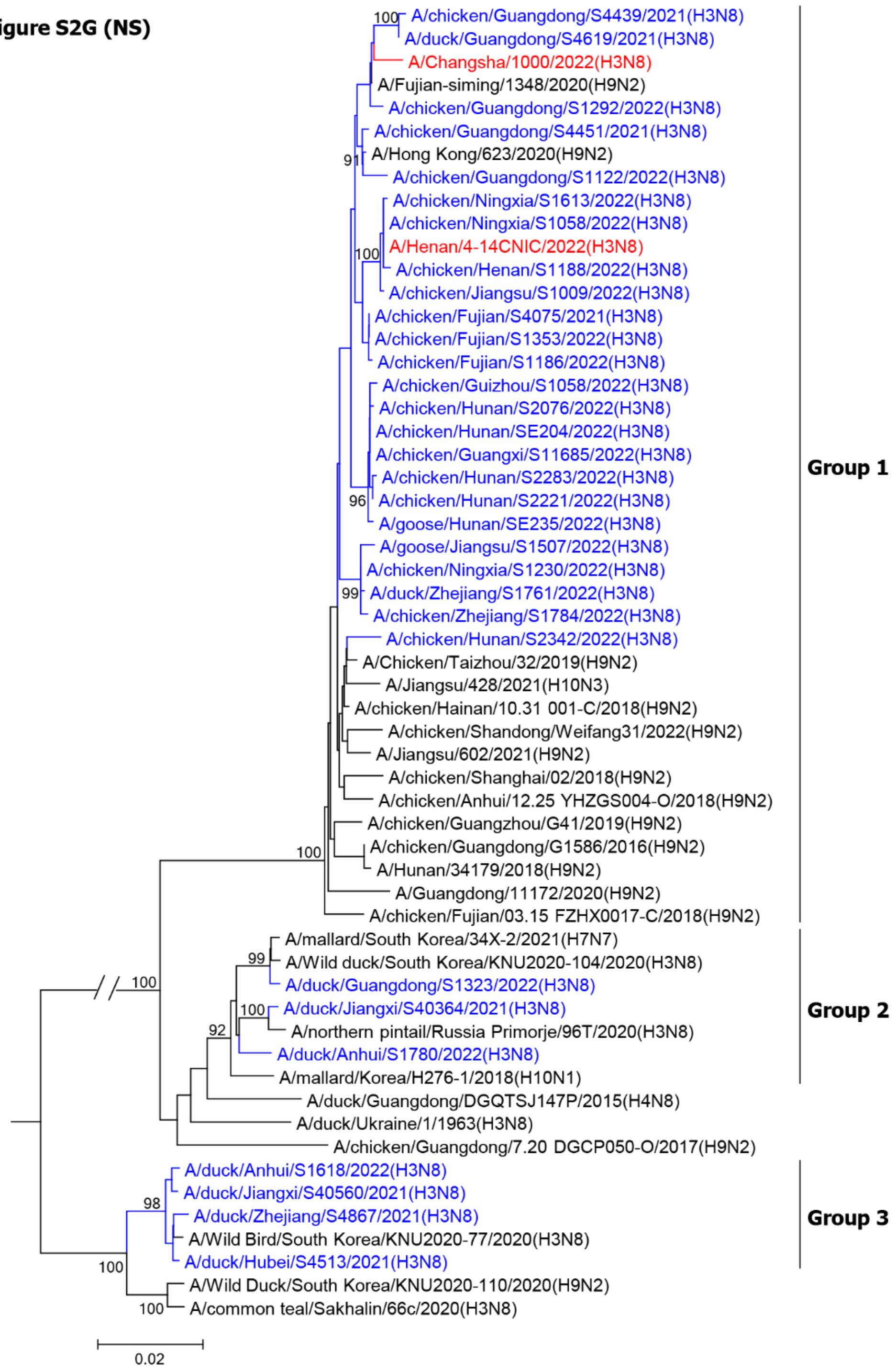

Supplementary Figure S3. Receptor-binding properties of H3N8 viruses isolated in China.

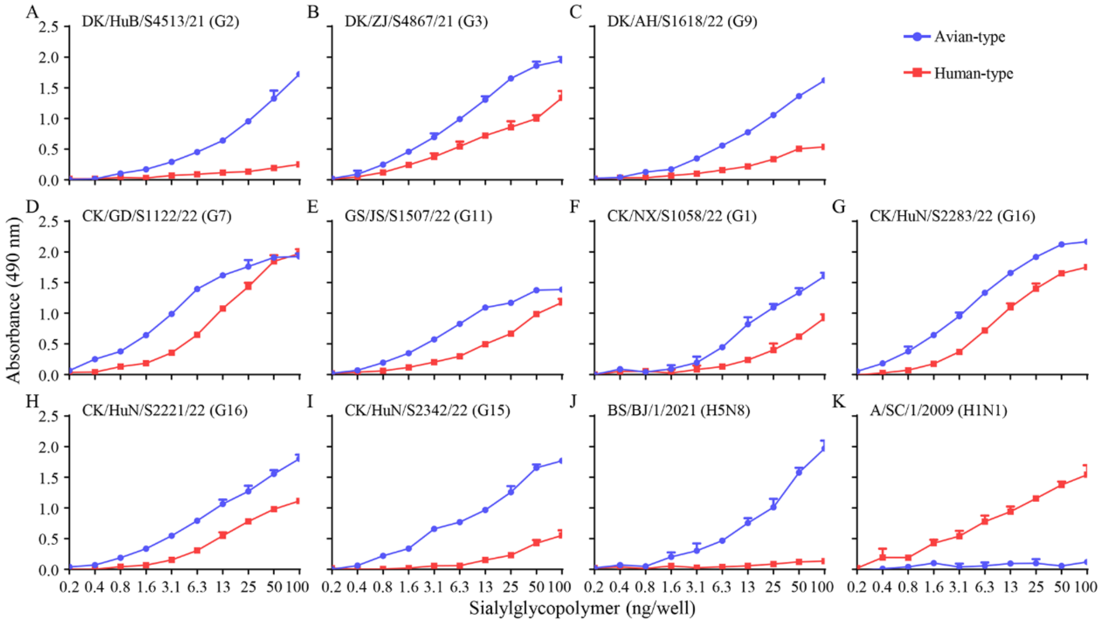

The data shown are the means of three repeats; the error bars indicate standard deviation. Two viruses, BS/BJ/1/2021 (H5N8) and A/SC/1/2009 (H1N1), that bind exclusively to α-2,3- and α-2,6-linked glycans, respectively, were used as controls.

Supplementary Table S1. Information on the haemagglutinin sequence of influenza A(H3N8) virus isolates used for phylogenetic analysis.

Segment ID	Segment	Country	Collection date	Isolate name	Originating laboratory	Submitting laboratory	Authors
EPI1330330	HA	Bangladesh	2016-Jul-22	A/duck/Bangladesh/20D677/2016	International Centre for Diarrhoeal Disease Research	Centers for Disease Control and Prevention	Yang, G.; Chowdury, S; Hodges, E; Rahman, M.Z.; Jang, Y.; Hossain, M.E.; Jones, J.; Stark, T.; Di, H.; Cook, P.W.; Ghosh, S.; Azziz-Baumgartner, E.; Barnes, J.; Wentworth, D.; Kennedy, E.; Davis, C.T.
EPI660359	HA	China	2015-Apr-22	A/duck/Guangdong/04.22 DGCP083P/2015	NA	Institute of Microbiology, Chinese Academy of Sciences	NA
EPI1590264	HA	China	2015-Apr-9	A/EN/Hunan/37939/2015	WHO Chinese National Influenza Center	WHO Chinese National Influenza Center	Shumei Zou, Jing Tang, Ye Zhang, Xiyan Li, Yao Meng, Xiang Zhao, Lei Yang, Yuelong Shu, Dayan Wang
EPI1489636	HA	China	2018-Nov-21	A/chicken/Guangdong/F117/2018	South China Agricultural University	South China Agricultural University	NA
EPI2035832	HA	China	2022-May-11	A/Changsha/1000/2022	Changsha center for disease control and prevention	Changsha Disease Prevention and Control Center	Huang, zheng; Ou, xinhua; Yang, Rengui
EPI2026173	HA	China	2022-Apr-14	A/Henan/4-14CNIC/2022	Henan provincial center for disease control and prevention	WHO Chinese National Influenza Center	NA
EPI1931674	HA	Korea, Republic of	2020-Oct-7	A/Wild duck/South Korea/KNU2020104/2020	NA	NA	Tiwari,I.; Tuong,H.T.; Sung,H.W.; Yeo,S.J.; Park,H.
EPI1931666	HA	Korea, Republic of	2020-Oct-5	A/Wild Bird/South Korea/KNU202077/2020	NA	NA	Tiwari,I.; Tuong,H.T.; Sung,H.W.; Yeo,S.J.; Park,H
EPI1903591	HA	Korea, Republic of	2019-Oct-5	A/Mallard/South Korea/KNU2019-50/2019	NA	NA	Indira,T.; Bao,D.T.; Vui,H.T.; Park,H.; Yeo,S.J.; Sung,H.W.

EPI1903918	HA	Korea, Republic of	2019-Oct-28	A/Green-winged teal/South Korea/KNU201957/2019	NA	NA	Indira,T.; Bao,D.T.; Vui,H.T.; Park,H.; Yeo,S.J.; Sung,H.W.
EPI1657083	HA	Russian Federation	2019-Sep-15	A/Common Teal/Russia Primorje/94/2019	Research Institute of Experimental and Clinical Medicine	National Institute of Animal Health	Mine, J.; Uchida, Y.; Saito, T.; Shestopalov, A.; Dubovitskiy, N.; Sobolev, I.; Derko, A.; Alekseev, A.; Sharshov, K.
EPI1847527	HA	Russian Federation	2020-Sep-8	A/common teal/Sakhalin/66c/2020	Research Institute of Experimental and Clinical Medicine	National Institute of Animal Health	Mine, J.; Uchida, Y.; Saito, T.; Shestopalov, A.; Dubovitskiy, N.; Sobolev, I.; Derko, A.; Alekseev, A.; Sharshov, K.
EPI1657099	HA	Russian Federation	2019-Oct-27	A/Northern Pintail/Russia_Primorje/298/2019	Research Institute of Experimental and Clinical Medicine	National Institute of Animal Health	Mine, J.; Uchida, Y.; Saito, T.; Shestopalov, A.; Dubovitskiy, N.; Sobolev, I.; Derko, A.; Alekseev, A.; Sharshov, K.
EPI1847535	HA	Russian Federation	2020-Sep-8	A/common teal/Sakhalin/81c/2020	Research Institute of Experimental and Clinical Medicine	National Institute of Animal Health	Mine, J.; Uchida, Y.; Saito, T.; Shestopalov, A.; Dubovitskiy, N.; Sobolev, I.; Derko, A.; Alekseev, A.; Sharshov, K.
EPI1850029	HA	Russian Federation	2020-Aug-29	A/mallard/Novosibirsk region/3265k/2020	Research Institute of Experimental and Clinical Medicine	National Institute of Animal Health	Mine, J.; Uchida, Y.; Saito, T.; Shestopalov, A.; Dubovitskiy, N.; Sobolev, I.; Derko, A.; Alekseev, A.; Gluschenko, A.; Sharshov, K.
EPI1358860	HA	Russian Federation	2018-Sep-15	A/teal/Dagestan/23d/2018	Research Institute of Experimental and Clinical Medicine	National Institute of Animal Health	Yoshida,E.; Mine, J.; Uchida, Y.; Saito, T.; Shestopalov, A.; Dubovitskiy, N.; Sobolev, I.; Derko, A.; Alekseev, A.; Murashkina, T.; Sharshov, K.
EPI1352470	HA	Russian Federation	2018-Sep-28	A/mallard/Novosibirsk region/957k/2018	Research Institute of Experimental and Clinical Medicine	National Institute of Animal Health	Yoshida,E.; Mine, J.; Uchida, Y.; Saito, T.; Shestopalov, A.; Dubovitskiy, N.; Sobolev, I.; Derko, A.; Alekseev, A.; Sharshov, K.
EPI1333763	HA	Russian Federation	2018-Oct-19	A/gadwall/Chany/893/2018	State Research Center of Virology and Biotechnology	State Research Center of Virology and Biotechnology	Natalia,Goncharova; Ivan,Susloparov; Natalia,Kolosova; Alexey,Danilenko; Juliya,Bulanovich; Vasiliy,Marchenko; Alexander,Ryzhikov
EPI966605	HA	United States	2012-Oct-5	A/northern pintail/Alaska/679/2012	NA	NA	Reeves,A.B.; Ramey,A.M.; Hall,J.S.
EPI729601	HA	United	2014-Sep-29	A/northern	NA	NA	Ramey,A.M.; Reeves,A.B.; TeSlaa,J.L.;

		States		pintail/Alaska/523/2014			Nashold,S.; Donnelly,T.;Bahl,J.; Hall,J.S.; Donnelly,T.F.
EPI973471	HA	United States	2015-Sep-11	A/green-winged teal/Alaska/UGAI15-6400/2015	NA	NA	Reeves,A.B.
EPI486122	HA	United States	2012-Jul-9	A/mallard/California/2743/2012	NA	NA	NA
EPI585221	HA	United States	2013-Feb (Day unknown)	A/redhead/Ohio/13OS363/2013	NA	NA	Bowman,A.; Nelson,S.; Sreevatsan,S.; Suwannakarn,K.; Slemons,R
EPI1769107	HA	United States	2018 (Month and day unknown)	A/Mallard/Ohio/18OS4675/2018	NA	NA	Killian,M.L.; Franzen,K.; Camp,P.; Stuber,T.;Robbe-Austerman,S.; Lauterbach,S.; McBride,D.; Nolting,J.; Bowman,A.
EPI1772918	HA	United States	2018-Sep-10	A/mallard/Minnesota/MN18-WB1065A/2018	NA	NA	Ramey,A.M.; Stallknecht,D.E.; Reeves,A.B.
EPI1772806	HA	United States	2018-Sep-11	A/mallard/Alaska/AK18-WB1-048A/2018	NA	NA	
EPI1154377	HA	United States	2016-Oct-15	A/American wigeon/Ohio/16OS3665/2016	NA	NA	Killian,M.; Franzen,K.; Camp,P.; Stuber,T.; Robbe-Austerman,S.; Lauterbach,S.; Nolting,J.; Bowman,A.
EPI735313	HA	United States	2014-Sep-4	A/mallard/Ohio/14OS0985/2014	NA	NA	Das,S.R.; Halpin,R.A.; Lin,X.; Simenauer,A.; Akopov,A.; Fedorova,N.; Puri,V.; Stockwell,T.; Amedeo,P.; Bishop,B.; Katzel,D.; Schobel,S.; Shrivastava,S.; Nolting,J.; Bao,Y.; Sanders,R.; Zhdanov,S.; Kiryutin,B.; Lipman,D.J.; Tatusova,T.; Slemons,R.; Bowman,A.

NA: not available.

Supplementary Table S2. Amino acids detected in the H3N8 viruses contribute to increased affinity to the human-type receptor, virulence in mammals, and resistance to amantadine[a].

Genotype (Number of avian strains)	Amino acids in HA that increase affinity to human-type receptors		Amino acids that increase the virulence of avian influenza viruses in mammals											Amino acids in M2 that increase the resistance to amantadine	
			PB2					PA				NS1			
	193N	228S	292V	588V	598I/T	627K/V	702R	63I	330V	356R	409N	42S	106M	27A	31N
G1 (6)	6	/	6	6	/	/	6	6	/	6	6	4	3	/	6
G2 (1)	/[b]	/	1	/	1 (T)	/	/	/	/	/	/	1	/	/	/
G3 (1)	1	/	/	/	1 (T)	/	/	/	/	/	/	1	/	/	/
G4 (1)	1	/	/	/	1 (T)	/	/	/	/	/	/	1	/	/	/
G5 (1)	1	/	1	/	1 (T)	/	/	/	/	/	/	1	/	/	/
G6 (1)	1	/	1	1	1 (I)	/	1	1	/	1	1	1	1	/	1
G7 (6)	6	/	6	6	/	/	6	6	/	6	6	5	2	1	6
G8 (1)	1	/	/	/	1 (T)	/	/	/	/	/	1	1	/	/	/
G9 (1)	1	/	1	/	1 (T)	/	/	/	/	/	/	1	/	/	/
G10 (1)	/	/	1	/	1 (T)	/	/	/	/	/	/	1	/	/	/

G11 (2)	2	/	2	2	2 (I)	/	/	/	2	2	2	1	1	/	2
G12 (3)	3	/	3	3	2 (I)	/	/	3	/	3	3	3	/	/	3
G13 (1)	1	/	1	1	/	/	1	1	/	1	1	1	/	/	1
G14 (1)	1	/	1	/	1 (T)	/	/	/	/	/	/	1	/	/	/
G15 (1)	1	/	1	1	/	1 (V)	/	1	/	1	1	1	1	/	1
G16 (2)	2	/	2	2	/	2 (V)	/	2	/	/	2	2	/	/	2
G17 (1)	1	/	1	1	/	/	1	1	/	1	1	1	/	/	1
A/Henan/4–14CNIC/2022	1	G/S[c]	1	1	/	1 (K)	1	1	/	1	1	1	/	/	1
A/Changsha/1000/2022	1	/	1	1	/	1 (V)	/	1	/	1	1	1	/	/	1
Total	31	1	30	25	5 (I), 8 (T)	1 (K), 4 (V)	16	23	2	23	26	29	8	1	25

[a]Several amino acid residues, including155T and 225G in HA, 309D, 431M, and 504V in PB2, 622G in PB1, 383D, 550L, and 639T in PA, 286A and 437T in NP, and 30D, 43M, and 215A in M1, are highly conserved and present in all of these viruses, and therefore they are not shown in the table.

[b]No such mutation;

[c]Both amino acids were detected at position 228 in HA according to the sequence information.
